# Age-related micro-RNA abundance in individual *C. elegans*

**DOI:** 10.18632/aging.100564

**Published:** 2013-06-12

**Authors:** Mark Lucanic, Jill Graham, Gary Scott, Dipa Bhaumik, Christopher C. Benz, Alan Hubbard, Gordon J. Lithgow, Simon Melov

**Affiliations:** ^1^ Buck Institute for Research on Aging, 8001 Redwood Boulevard, Novato, CA 94945, USA; ^2^ Division of Biostatistics, University of California, Berkeley, Berkeley, CA 94720, USA

**Keywords:** Aging, miRNA, DAF-16, Insulin/IGF, mir-71, expression profiling

## Abstract

Non-coding small RNAs of the micro-RNA class (miRNA) are conserved regulators of gene function with a broad impact on biological processes. We screened miRNA levels for age-related changes in individual worms and investigated their influence on the lifespan of the nematode C. elegans. We measured the abundance of 69 miRNAs expressed in individual animals at different ages with over thirty five thousand discrete quantitative nano-fluidic polymerase chain reactions. We found that miRNA abundance was highly variable between individual worms raised under identical conditions and that expression variability generally increased with age. To identify expression differences associated with either reproductive or somatic tissues, we analyzed wild type and mutants that lacked germlines. miRNAs from the mir-35-41 cluster increased in abundance with age in wild type animals, but were nearly absent from mutants lacking a germline, suggesting their age-related increase originates from the germline. Most miRNAs with age-dependent levels did not have a major effect on lifespan, as corresponding deletion mutants exhibited wild-type lifespans. The major exception to this was mir-71, which increased in abundance with age and was required for normal longevity. Our genetic characterization indicates that mir-71 acts at least partly in parallel to insulin/IGF like signals to influence lifespan.

## INTRODUCTION

An understanding of the mechanisms that modulate the lifespan of eukaryotes has great potential to promote human health. Aging is the single largest risk factor for many debilitating diseases, including neurodegenerative diseases such as Alzheimer's and Parkinson's disease, as well as cardiovascular diseases, and cancer. An inherently important problem for aging research lies in understanding why some people age well and remain disease free, while others age poorly, and incur increased risk for age-related disease. It is clear that there are genetic factors which individually or collectively contribute to age-related disease via altered function, however, relative maintenance of health with increased age alone does not equate with the absence of age-related disease. In the round worm Caenorhabditis elegans, genetically identical animals exhibit large differences in their lifespan with associated declines in motor skills, and pathogen resistance [1-4]. We have previously shown that aging behavioral phenotypes in individual worms are associated with statistically significant changes in gene expression [5]. We hypothesized that the distinct age dependent gene expression profiles that exist between genetically identical individuals are likely to be mediated through variations in gene regulatory networks. miRNAs represent likely candidates for mediating some of this variation in expression as they are known modulators of gene expression, which have been shown to act to facilitate the robustness of such networks [6].

miRNAs are conserved and highly abundant post-transcriptional regulators of gene expression [7-10].

Since their relatively recent discovery [11, 12] miRNAs have been implicated in a remarkable number of diverse biological processes. Recent work has shown that miRNAs are associated with many physiological processes correlated with aging including cellular senescence [13], stem cell proliferation [14, 15] and IGF (insulin-like growth factor) expression [16]. These results suggest key roles for miRNAs in modulating aging, and aging related phenotypes. Additional studies have reported that the expression of miRNAs are themselves altered during normal aging in C. elegans as well as in humans [17-20]. Collectively, this suggests that miRNAs could play a role in organismal aging. Recent reports have demonstrated that in C. elegans miRNAs modify lifespan [17, 21, 22], although the mechanisms and direct biological targets of the miRNAs remain elusive. Insulin/IGF like signaling is a key modulator of lifespan through its control of a transcriptional regulatory network and represents a potential target for miRNAs to modify lifespan.

Insulin/IGF like signaling modulates lifespan in many model organisms ranging from worms to vertebrates [23-27]. Recent human genetic epidemiological studies suggest that insulin or IGF-like signaling is associated with human aging traits including longevity [28, 29]. Hence the regulatory mechanisms of insulin signaling in model organisms are likely to be important in understanding genetic modulators of human lifespan and healthy aging. In the nematode C. elegans insulin/IGF like signaling is controlled through the sole insulin/IGF type receptor DAF-2, whose activity represses the FOXO transcription factor DAF-16. In *C. elegans* decreased insulin/IGF like signaling results in extended lifespan and increased stress resistance through activation of DAF-16 and its transcription network.

Here we present expression and functional evidence that miRNA expression levels vary between individuals and that at least one of these miRNAs has a dramatic influence on aging. There is considerable phenotypic variation in aging animals, some of which may be attributable to miRNA-regulated protein synthesis. We have utilized a high throughput nano-fluidic PCR approach to analyze individual worm miRNA expression profiles, in order to assess age-dependent differences and between-worm variance in miRNA abundance. We report that certain miRNAs exhibit strong age-specific miRNA changes in abundance and individual variation in miRNA levels. We find that few of these miRNAs strongly influence lifespan by themselves. The exception to this finding was *mir-71* which had a major effect on longevity. Interestingly, we determine that at least part of *mir-71* effect on wild type lifespan is independent of insulin/IGF like signaling.

## RESULTS

### Nano-fluidic quantitative polymerase chain reaction of miRNA samples from individual worms

**Figure 1:**
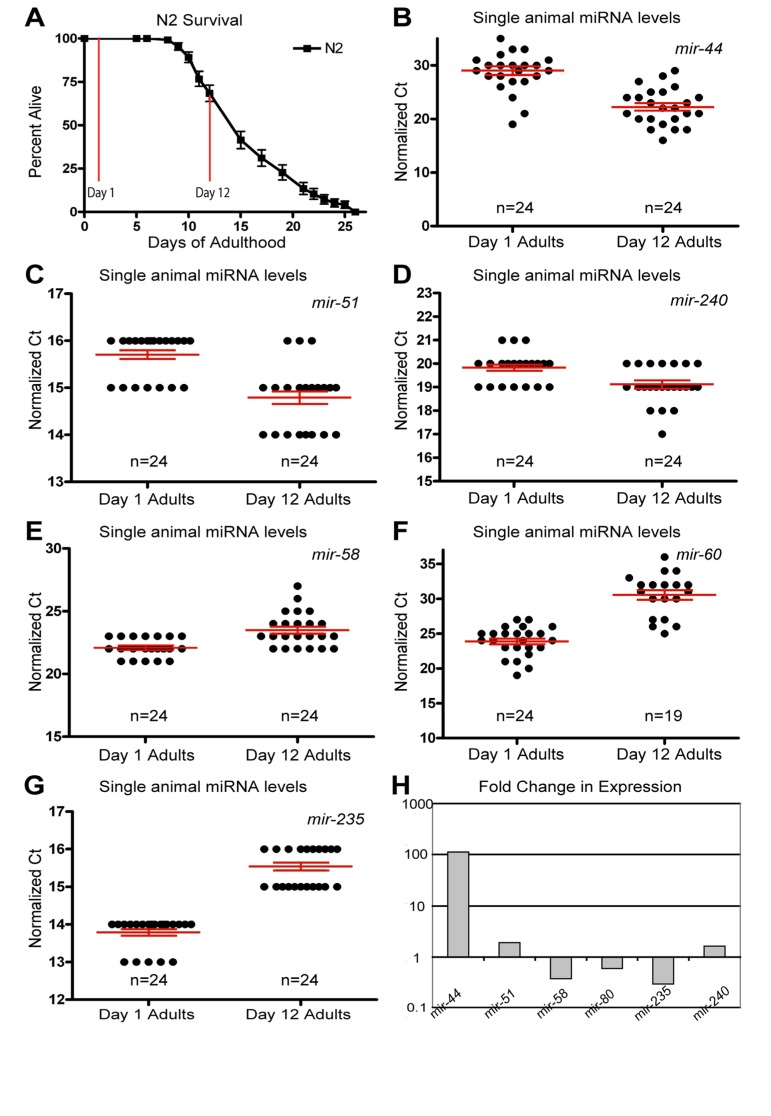
Dynamic changes of miRNAs with age (**A**) Survivorship of a population of N2 (wild type) animals, from which individuals were harvested for analysis at Day 1 and Day 12 of adulthood. (**B-G**) Normalized Ct values describing miRNA abundance in individual worms (each point represents data from a single animal) at two ages are shown for *mir-44* (B), *mir-51* (**C**), *mir-240* (**D**), *mir-58* (**E**), *mir-60* (**F**), and *mir-235* (**G**). (**H**) Graphical representation of the fold change in miRNA abundance between young and old animals. In all graphs with error bars they indicate the standard error of the mean. In individual worm graphs each point is the average from 2-3 technical replicates.

To examine age-related miRNA expression changes we used the quantitative polymerase chain reaction (qPCR) to assay a panel of 69 miRNAs in RNA samples collected from 48 C. elegans animals. We used individual worms to assay the miRNA levels, an approach which has previously been used in the context of gene expression profiling via microarrays [5, 30]. Such an approach captures variation between animals with age, as it screens individuals as opposed to most conventional methods which analyze populations containing large numbers of pooled animals. Individual wild type (N2; Bristol strain) animals were examined for expression of each miRNA simultaneously using dynamic integrated nano-fluidic circuits (Fluidigm inc.) with Taqman qPCR assays (ABI) specific for each miRNA [31]. We first tested for expression of the miRNAs in 24 young (hermaphrodites on their first day of adulthood) and 24 old (hermaphrodites on their twelfth day of adulthood) animals. Young adult animals were non-gravid, while old animals were post-reproductive and were collected while the population was undergoing a high mortality rate (Figure 1A). We found that 45 miRNAs were consistently detected (in more than half of the animals) while 43 miRNAs were consistently detected in old animals (Supplemental Table 1). Of the 43 miRNAs that were consistently detected in both young and old animals, slightly more than half (56%) decreased in abundance with age, demonstrating that amongst the miRNAs we assayed, there is a trend towards most miRNAs decreasing in expression with age. As we had determined the levels of miRNAs in individual animals, we next examined the variation in abundance of each miRNA between individual worms for both young and old animals (standard deviation of the population from the mean Ct (cycle threshold)). There is a generalized increase in the variation of miRNA abundance with age, consistent with a generalized increase in stochastic dysregulation. Specifically we found that 68% of the miRNAs showed an increase in variation between individual worms with age, while if we only counted the miRNAs which were detected in all 48 animals then 74% of these showed an increase in their variation with age (Supplemental Table 1). This suggests that variability of miRNA expression generally increases with age. This increase in variability may be in part due to stochastic cell and tissue loss in aged animals [2, 32-34].

**Figure 2:**
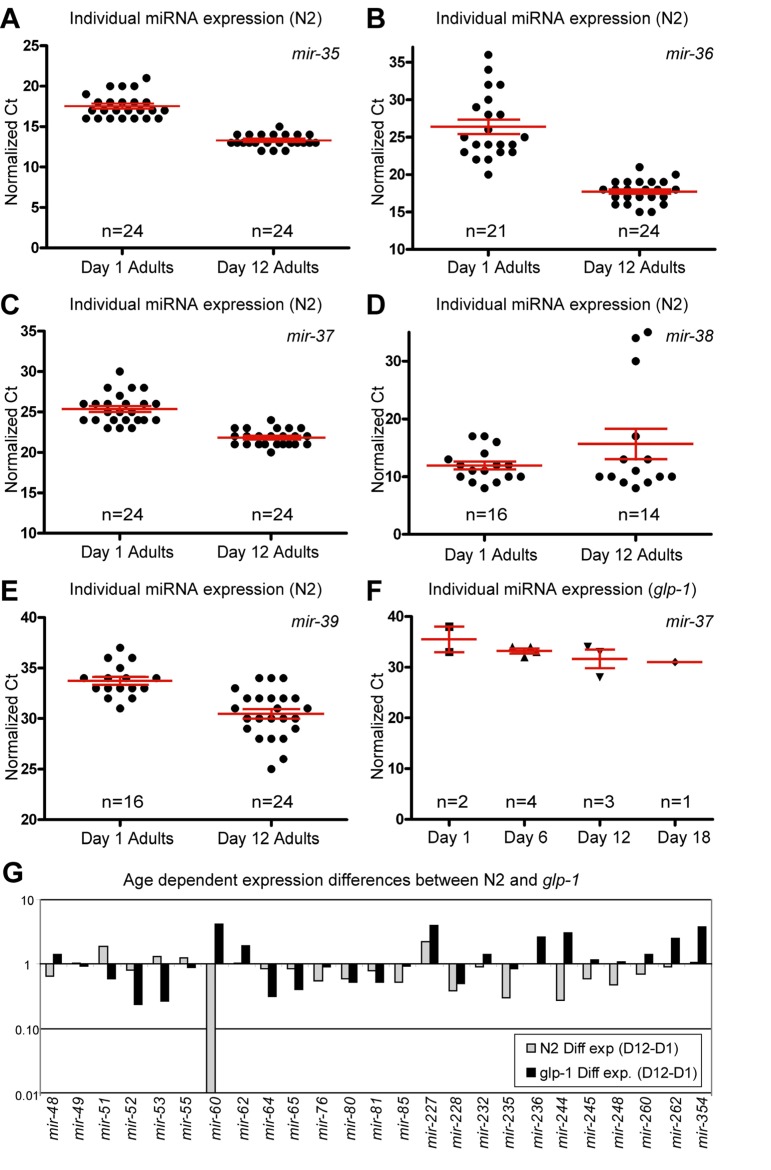
Presence of the germline alters miRNA abundance (**A-F**) Normalized Ct values demonstrating that miRNA expression from the *mir-35-41* cluster is dramatically different in N2 animals with age but is virtually absent, at all ages examined, in *glp-1* germline-less animals. In N2 animals, *mir-35* (**A**), *mir-36* (B) and *mir-37* (**C**) are expressed at much higher levels in old animals, while *mir-38* (D) has a median value that is higher in aged animals. *mir-39* (**E**) is also dramatically higher in aged animals. In *glp-1* mutants only *mir-37* (**F**) was detected, and its levels were much lower than in wild type animals. (**G**) Graphical representation of the difference between miRNA abundance in old and young worms, for both wild type and germline-less mutants. The values graphed are the average values at the old age minus the averaged values at the young age for each of the two genotypes with log2 transformation to describe the fold change (i.e. 2^-(average Ct old - average Ct young)). In all graphs with error bars they indicate the standard error of the mean. In individual worm graphs each point is the average from 2-3 technical replicates.

Analysis of the expression profiles in single worms lead to the identification of miRNAs which had significantly altered levels between young and old animals (Figure 1 and Table 1). Some miRNAs that showed altered levels with age had higher levels later in life. Of these, the most statistically significant increases were observed for miRNAs; mir-51, mir-44, mir-240 and the members of the mir-35-41 cluster (Figure 1B-D and Figure 2A-E). We also identified miRNAs whose levels decreased significantly with age; among these mir-235, mir-60 andmir-58 were identified as those being consistently detected and showing the most significant decrease with age (Figure 1E-G). Other than the mir-35 family members all of these miRNAs showed an increase in variation with age (Supplemental Table 1). To test these results with an independent approach we quantified green fluorescent protein (GFP) expression in aged worm strains carrying promoter-GFP fusions for the age dependent hits mir-1 (Supplemental Figure 1), mir-60 (Supplemental Figure 2) and mir-228 (Supplemental Figure 3). In agreement with our biochemical results, these strains showed decreased expression of GFP with age.

**Table 1:** miRNAs showing age-dependent abundance in wild type with statistical ranking

Gene	Diff Exp	Raw p Value	Low 95 CI	Up 9 5CI	FWER
***mir-235***	**0.30**	**6.51E-22**	**0.26**	**0.35**	**3.1E-20**
***mir-35***	**18.09**	**1.68E-17**	**11.79**	**27.74**	**8.1E-16**
***mir-37***	**12.15**	**2.38E-11**	**6.92**	**21.32**	**1.1E-09**
***mir-36***	**415.74**	**3.34E-11**	**107.90**	**1601.92**	**1.6E-09**
***mir-60***	**0.01**	**1.08E-09**	**0.00**	**0.03**	**5.2E-08**
***mir-51***	**1.82**	**2.45E-08**	**1.53**	**2.17**	**1.2E-06**
***mir-44***	**106.35**	**9.59E-08**	**24.88**	**454.66**	**4.6E-06**
***mir-39***	**9.84**	**9.90E-06**	**4.07**	**23.78**	**4.8E-04**
***let-7***	**0.04**	**1.39E-05**	**0.01**	**0.14**	**6.7E-04**
***mir-58***	**0.38**	**4.30E-05**	**0.25**	**0.58**	**2.1E-03**
***mir-1***	**0.43**	**6.39E-05**	**0.29**	**0.62**	**3.1E-03**
***mir-80***	**0.62**	**1.30E-04**	**0.50**	**0.78**	**6.3E-03**
***mir-240***	**1.69**	**1.77E-04**	**1.31**	**2.17**	**8.5E-03**
***mir-81***	**0.76**	**4.65E-04**	**0.65**	**0.87**	**2.2E-02**
***mir-228***	**0.40**	**4.99E-04**	**0.25**	**0.65**	**2.4E-02**
***mir-70***	**0.16**	**7.68E-04**	**0.06**	**0.42**	**3.7E-02**
*mir-55*	1.26	1.05E-02	1.06	1.50	5.0E-01
*mir-82*	0.82	1.27E-02	0.71	0.95	6.1E-01
*mir-64*	0.83	1.45E-02	0.71	0.96	7.0E-01
*mir-71*	1.34	1.63E-02	1.06	1.68	7.8E-01
*mir-234*	0.36	1.93E-02	0.16	0.81	9.2E-01
*mir-248*	0.46	2.53E-02	0.24	0.89	1.0E+00
*mir-244*	0.28	3.51E-02	0.09	0.87	1.0E+00
*mir-54*	1.25	5.39E-02	1.00	1.56	1.0E+00

The table presents statistical values for miRNAs determined to have age dependent abundance in wild type. Diff Exp, is the approximate ratio of expression comparing Young to Old animals (11 day difference in age). The raw p-value is for the null hypothesis that the Diff exp Ratio = 1 (based on robust inference - see supplemental information) and the associated 95% CI of the ratio. Additionally, we present an adjusted p-value based on family wise error rate (FWER) using a Bonferroni adjustment for multiple comparisons).

### High expression of *mir-35* family members in aged animals originated in the post-reproductive germline

Some of the largest and most significant changes in levels of miRNAs between first day adults and post-reproductive adults came from members of the mir-35 miRNA family and specifically came from the mir-35-41 gene cluster (Figure 2A-E and Table 1). We found that the mir-35 cluster members in our set of assays (mir-35-39) showed a dramatic increase in abundance with age. The only exception being *mir-38* which included three outlier signals that shifted the overall mean towards a net decrease in levels, while still having a median increase in abundance with age (Figure 2D and Supplemental Table 1). mir-35 family members are redundantly required for viability and are highly expressed in developing embryos [35, 36]. The *mir-35-41* gene cluster is also co-transcriptionally regulated [36] and it has been reported that expression from the mir-35-41 cluster originated in the germline and was likely to be mainly found in oocytes and embryos [35]. These results, along with our previous observation of the DNA increase in the post-reproductive germline [32, 33] led us to speculate that we may be observing an increase in expression from dysregulated genomes of immediate germline descent. Indeed many of the profiling candidates we identified as having altered abundance with age may be a result of changes in germline expression due to the massive increase in germline DNA copy number with age.

**Figure 3:**
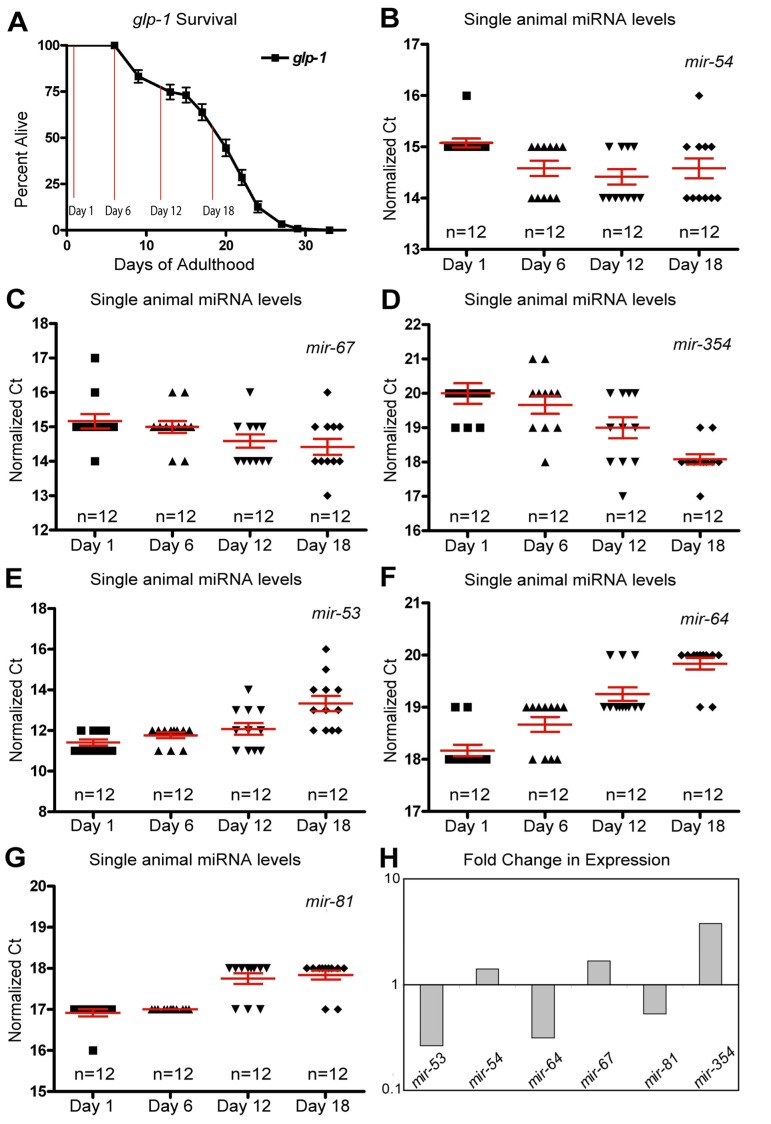
Dynamic changes of miRNAs with age in long-lived germline ablated animals (**A**) Survivorship of a population of N2 (wild type) animals, from which individuals were harvested for analysis at Day 1, Day 6, Day 12 and Day 18 of adulthood. (**B-G**) miRNA levels in individual worms (columns) at four time points are shown for *mir-54* (**B**), *mir-67* (**C**), *mir-354* (**D**), *mir-53* (**E**), *mir-64* (**F**), and *mir-81* (**G**). (**H**) Graphical representation of the fold change in miRNA abundance between young and old animals using averaged values. In all graphs with error bars they indicate the standard error of the mean. In individual worm graphs, each point is the average from 2-3 technical replicates.

To test the contribution of the germline on miRNA expression we performed qPCR analysis of miRNA levels, on germline-less animals. We chose a mutant strain that allowed for genetic ablation of the germline during development. glp-1(e2141) mutants are temperature sensitive sterile and at the restrictive temperature lack a germline due to the inability of the germline stem cells to remain mitotically active. These animals are also long-lived in a DAF-16 and DAF-12 dependent manner [37], however they still respond to deficits in insulin/IGF like signaling with lifespan extension. To examine changes with age in animals lacking a germline, we collected individual *glp-1* mutants spanning 4 adult ages that included both young and old animals (Figure 3A). Interestingly we found that expression from the mir-35-41 gene cluster was nearly absent in animals that lacked a gonad. Of the *mir-35-41* cluster we only detected *mir-37* in the germline ablated animals, but only in 30% of the individual worm samples. The levels of *mir-37* we measured were much lower than those observed in wild type young adult animals (Figure 2F and compare Supplemental Tables 1 and 2). These results along with previous reports, suggests that the age-dependent increase in the *mir-35* group arises from high expression in the germline of post-reproductive animals.

### The presence of the germline altered the expression of several miRNAs

In addition to the mir-35-41 cluster there were other individual miRNAs whose expression profiles are distinctly different between wild type animals and germline-less glp-1 mutants. Of these, mir-51 showed a significant elevation in expression with aging in wild type animals (Figure 1C). However, in the germline ablated glp-1 mutant, *mir-51* expression tended to decrease with age (Figure 2G). One interpretation of this result is that mir-51 is expressed in both somatic and germline tissues but that the trend in its expression with age is inverted for the two tissues. Another miRNA expression profile that showed a dramatic difference between wild type and germline less mutants was mir-60, which showed a significant and profound decreased expression profile with age in wild type animals (Figure 1F). However, in mutants lacking a germline, mir-60 showed an average elevated expression in all of the ages tested relative to the first day adults (Figure 2G). This suggests that the germline normally functions to suppress somatic expression of mir-60, possibly implicating it in germline regulated somatic signaling cascades [37]. However, it is not absolutely required for the resulting lifespan extension observed from germline stem cell ablation (Supplemental Table 3).

As with the wild type miRNA expression profiles, analysis of the glp-1 profiles indicated that many miRNAs show altered abundance with age (Figure 3B-F and Table 2). Nine miRNAs showed robustly significant age-dependent levels (Table 2). The most statistically significant miRNAs showing increased expression with age were *mir-354* and *mir-240*, while *mir-54* and *mir-67* trended towards significance (Figure 3B-D, H and Table 2). In the germline ablated animals most of the statistically significant changes we observed showed a decrease in levels with age (78%). Of these *mir-64*, *mir-81*, *mir-65*, *mir-53*, *mir-58* were all significantly down-regulated in aged animals (Figure 3E-H and Table 2). Interestingly, and analogous to our results with wild type animals, we found that the variation in individual worm miRNA levels tended to increase with age, as 63% of miRNAs assayed showed an increase in variation. However, if we only enumerated those miRNAs that were detected in all individuals 79% of miRNAs showed an increase in variability across individuals with age (Supplemental Table 2). Collectively these results indicate that the variation of miRNA between individuals is not merely an artifact of post-reproductive germline abnormalities.

**Table 2:** miRNAs showing age-dependent abundance in *glp-1* with statistical ranking

Gene	Diff Exp	Raw p Value	Low 95 CI	Up 95 CI	FWER
***mir-64***	**0.52**	**1.8E-15**	**0.46**	**0.58**	**8.9E-14**
***mir-81***	**0.63**	**1.9E-11**	**0.57**	**0.70**	**9.4E-10**
***mir-354***	**2.15**	**8.0E-07**	**1.65**	**2.79**	**4.0E-05**
***mir-80***	**0.66**	**4.6E-06**	**0.56**	**0.77**	**2.3E-04**
***mir-65***	**0.53**	**5.7E-06**	**0.42**	**0.68**	**2.9E-04**
***mir-53***	**0.46**	**2.4E-05**	**0.33**	**0.64**	**1.2E-03**
***mir-240***	**2.01**	**3.6E-05**	**1.49**	**2.71**	**1.8E-03**
***mir-58***	**0.34**	**1.8E-04**	**0.20**	**0.57**	**8.9E-03**
***mir-235***	**0.84**	**3.4E-04**	**0.78**	**0.92**	**1.7E-02**
*mir-228*	0.64	1.4E-03	0.50	0.83	6.9E-02
*mir-82*	0.70	4.2E-03	0.55	0.88	2.1E-01
*mir-54*	1.32	5.3E-03	1.10	1.59	2.6E-01
*mir-52*	0.45	5.4E-03	0.26	0.77	2.7E-01
*mir-51*	0.81	8.6E-03	0.70	0.94	4.3E-01
*mir-67*	1.38	1.3E-02	1.08	1.76	6.6E-01
*mir-1*	0.64	1.8E-02	0.44	0.91	8.9E-01
*mir-273*	1.26	2.4E-02	1.04	1.52	1.0E+00
*mir-84*	1.06	2.7E-02	1.01	1.12	1.0E+00
*mir-70*	0.20	4.2E-02	0.05	0.87	1.0E+00
*mir-71*	1.41	5.2E-02	1.01	1.98	1.0E+00

The table presents statistical values for miRNA determined to have age dependent abundance in *glp-1* animals. Diff Exp, is the approximate ratio of expression comparing Young to Old animals (11 day difference in age). The raw p-value is for the null hypothesis that the Diff exp Ratio = 1 (based on robust inference - see supplemental information) and the associated 95% CI of the ratio. Additionally, we present an adjusted p-value based on family wise error rate (FWER) using a Bonferroni adjustment for multiple comparisons).

### Most miRNAs exhibited no or relatively minor influences on lifespan

**Figure 4:**
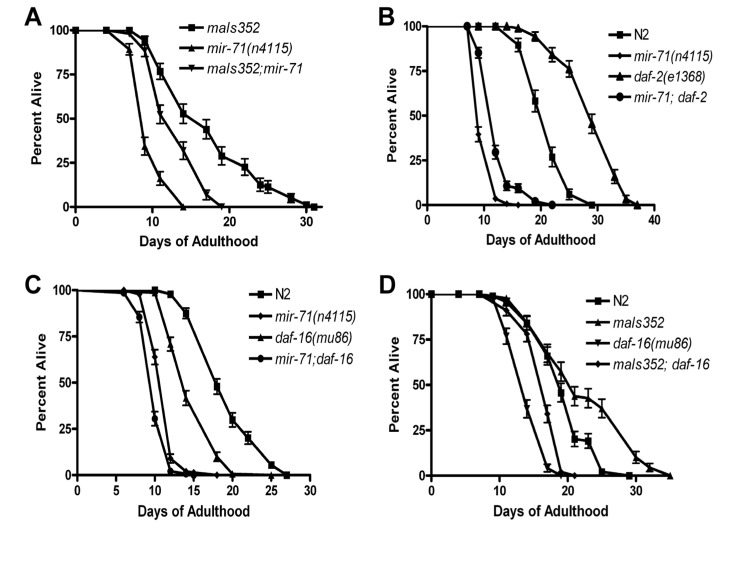
*mir-71* acts at least partly in parallel to the IIS pathway (**A**) The integrated transgene *maIs352[Pmir-71::mir-71::GFP]* partially rescues the lifespan of *mir-71* null mutants. (**B**) The longevity phenotype of *daf-2(e1368)* is significantly suppressed by the presence of the *mir-71(n4115)* deletion. (**C**) *daf-16; mir-71* double null mutants show significant enhancement relative to the single mutants. (**D**) Over-expression of *mir-71* extends the lifespan of *daf-16* null mutants.

In order to test whether miRNAs displaying age-specific abundance modulate lifespan, we tested mutant strains for several of the candidate miRNAs. These mutants were directly assayed for their lifespan and were compared to wild type animals (Supplemental Table 3). Of these, we found that one mutant strain carrying a deletion resulting in the removal of several miRNAs led to a weak lifespan extension. Specifically the deletion nDf48 that removes mir-42-44 led to a small but significant lifespan extension. (Supplemental Table 3), suggesting that individually or collectively these miRNAs may inhibit longevity in wild-type animals. We have also identified single miRNA mutations that resulted in mild or inconsistent lifespan phenotypes. The mir-80 allele nDf53 had a weak but significantly extended lifespan (Supplemental Table 3), while the *mir-228(n4382)* mutant trended towards an extended lifespan (Supplemental Table 3). In addition to miRNA mutants that showed an extended lifespan, we identified miRNA mutants that had shortened lifespans. We found that the mir-71 deletion n4115 had a profoundly shortened lifespan compared to wild type (Figure 4 and Supplemental Table 3), while the *mir-60* deletion, *n4947* trended towards shortened lifespan but was not consistently significantly different from wild type (Supplemental Table 3).

To further investigate the role of miRNAs in modulating lifespan, we analyzed the effect of expressing the missing miRNAs directly in the miRNA mutants. If the miRNAs absence in the mutants was causative of the lifespan phenotype, then these transgenic animals should be rescued for that phenotype or otherwise show a modulation of lifespan. We tested several such miRNA mutants containing transgenes and found that several of these mutants which exhibited lifespan phenotypes, showed modulation of the lifespan phenotype when the corresponding transgene was present (Supplemental Figure 4 and Supplemental Table 3). These included *mir-1*, *mir-80* and *mir-228*. This further suggests that these miRNAs may influence the lifespan of the organism. We found that several of the miRNAs mutants that did not exhibit lifespan phenotypes (the *mir-64* cluster, *mir-81-82* and *mir-235*) also failed to show lifespan phenotypes when the miRNAs were added back (Supplemental Table 3). Collectively, these results indicate that *mir-1*, *mir-80* and *mir-228* may have a role in modulating lifespan.

### *mir-71* acts at least partly in parallel to insulin/IGF signals

We next investigated the mechanism underlying a specific miRNA's effect on lifespan. We focused on *mir-71* which when mutant led to robust and significant lifespan phenotype, demonstrating its requirement for a normal long life. We examined the spatial expression pattern of *mir-71* using a promoter-GFP fusion strain [36] (Supplemental Figure 4). As previously reported mir-71 is expressed from early embryogenesis through to adulthood [38]. In early development expression of GFP from the mir-71 promoter is restricted to the primordial intestine of the embryo, but after hatching appears to be expressed in most non-neuronal tissues (Supplemental Figure 4). As GFP expression was very pronounced in the pharynx (Supplemental Figure 4A-F) we addressed whether this tissue is important for *mir-71* function in lifespan modulation. We found that pharyngeal expression of mir-71 could partly rescue the lifespan of mir-71 null mutants (Supplemental Figure 4G). This suggests that the pharynx is an important mediator of mir-71 function and likely contains targets of the putative regulatory RNA. We also analyzed expression of *mir-71* under strong heterologous promoters and found they led to arrest and unstable extra-chromosomal arrays (data not shown). A heat shock specific promoter driven construct was more stable and exhibited little developmental delay. Transgenic worms over-expressing *mir-71* were found to be long-lived (Supplemental Figure 4H). This result demonstrates that *mir-71* is sufficient to extend lifespan.

Since over-expression from heterologous promoters led to developmental delay and loss of the transgene, we utilized a stably integrated over-expressor of *mir-71* for further genetic analysis. We chose the promoter-GFP fusion construct for *mir-71*, known as maIs352 [36], which was of particular interest since it contains the entire mir-71 sequence. Consistent with this strain containing, the entirety of*mir-71* under its own promoter as well as GFP, we observed over-expression of mir-71 in the strain (Supplemental Figure 4I). Importantly we found that the transgene partly rescued a mir-71 null mutant (Figure 4A and Supplemental Figure 4I). We conclude that functional mir-71 is required for animals to survive late into normal adulthood, and that mir-71 acts in a pro-longevity manner. Along with our expression profiling results demonstrating that mir-71 levels increase with age (Supplemental Table 1-2 and Supplemental Figure 6A-B), we suggest that the normal function of mir-71 is sufficient to extend the lifespan of the adult animal.

It has previously been reported that mir-71 mutants are short-lived and that over-expression of mir-71 extended lifespan [17]. It was further suggested that*mir-71* acts with insulin/IGF signaling to modulate the lifespan of *C. elegans*. We found this interesting as we had identified a putative *mir-71* binding site in the 3' UTR of the insulin/IGF type receptor, DAF-2 (Supplemental Figure 6C). DAF-2 is a modulator of lifespan, whose effects are attributed to its role in regulating the FOXO transcription factor DAF-16. In agreement with de Lencastre and colleagues we found that *mir-71* mutants shorten the lifespan of *daf-2* hypomorphic mutants (Figure 4B). Collectively these data suggested to us that *mir-71* may regulate expression of the *daf-2* mRNA directly. To determine whether *mir-71* regulates the expression of DAF-2 we generated GFP fusion constructs with either *daf-2* or control 3'UTRs. However, we were unable to detect significant GFP expression differences between these constructs in *mir-71* mutant or *mir-71* over-expressor backgrounds (data not shown). These data suggested that *mir-71* does not directly regulate the expression of DAF-2.

We next sought to test whether the lifespan effects of *mir-71* depend on a functional insulin/IGF pathway. DAF-16 is the major transcription factor downstream of DAF-2 and is required for the lifespan effects resulting from IIS in *C. elegans*. If *mir-71* acts solely with IIS, then the effects of *mir-71* should depend on functional DAF-16. We therefore tested the lifespan phenotype of mir-71; daf-16 double null mutants. We find that the mir-71(n4115); daf-16(mu86) double mutants are significantly shorter lived than either daf-16 or mir-71 mutants alone (Figure 4C). This result indicates that mir-71 acts in parallel to DAF-16 to modulate adult lifespan. This result is inconsistent with the finding of de Lencastre and colleagues who performed a similar analysis but with non-null *daf-16(RNAi)*. To further test whether*mir-71* acts independently of IIS we next tested whether DAF-16 is required for the lifespan extending effects of *mir-71* over-expression. We found that the mir-71 over-expressor, *maIs352* significantly extends the lifespan of daf-16 null mutants (Figure 4D). Collectively our data demonstrates that mir-71 acts at least partly in parallel to insulin/IGF type signaling to modulate lifespan in *C. elegans*.

## DISCUSSION

The identification of genes that modulate healthy lifespan is a major goal of research on aging. Detailed functional analysis of these genes will strengthen our understanding of the mechanisms through which organisms grow old, and maintain health. We reasoned that individual gene expression differences between young and old animals may contribute to age dependent phenotypes. To identify such genes we performed expression profiling and focused specifically on miRNAs, several of which showed altered levels with age (Tables 1-2). With single worm qPCR we also examined heterogeneity in miRNA expression between individual worms. We found that miRNA expression varies between individual animals and that this variation generally increases with age. It is tempting to speculate that this heterogeneity of expression contributes to the apparent stochasticity of individual lifespan for isogenic animals. Though our current results cannot confirm this, they do point to potential candidates. As the germline is known to contribute to lifespan determination, we also identified which candidates were likely to be expressed mainly in the germline (miRNAs whose levels dropped dramatically in germline ablated animals; e.g. *mir-35* family) or somatic tissues (miRNAs whose abundance changed little in germline ablated animals (many miRNAs including *mir-71* and *mir-80*). Finally, we identified which miRNAs showed increases in abundance in germline ablated animals (such as *mir-60*). The last case may be of particular interest for aging studies since these miRNA expression differences in *glp-1* long-lived mutants correlate well with other genes known to be involved in germline loss mediated lifespan extension. Additional studies will be required to tell whether these miRNAs play a role in this process. We also note the possibility that some of the miRNA expression profiles obtained in the *glp-1* background may be influenced by non-germline related effects of *glp-1*.

Our expression profiling of miRNAs from aging C. elegans can be qualitatively compared with previous reports presenting similar data obtained with different techniques of both collection (whole population samples) and detection (micro-array and next generation sequencing) [19, 20]. A qualitative comparison of these previously published results and our own demonstrates significant overlap between hits, for example all studies identified *let-7* and *mir-70* as decreasing with age, while the more recent study as well as the work presented here identified the *mir-35* family members, *mir-54* and *mir-71* as increasing with age. However, significant differences exist as well, possibly explained by technical differences in both collection and analysis. The qPCR data presented here has the benefit of being quantitative over a wide dynamic range as well as having high resolution, the potential to relate individual phenotypes to gene expression (single worm), and to capture the biological variance in the aging population through virtue of a high biological N. In comparison the previously reported micro-array and next generation sequencing data has the benefit of being much higher throughput in terms of number of targets assayed, although they were more limited in the number of biological replicates. These other methods also possess larger range of coverage for potential involvement of miRNAs in aging, while our study was limited to a smaller number of assays available for qPCR. The next generation sequencing method in particular has discovery based appeal since the probe based bias is not present as it is with microarray and qPCR experiments.

We identified multiple miRNAs in our profiling experiments that exhibited age-related changes in abundance, some of these were tested for direct effects on lifespan through mutant analysis. We surveyed over 25 miRNA mutants for effects on lifespan and identified only 1 (*mir-71*) that differed dramatically from wild type. Some mutants were found to have relatively small or inconsistent lifespan phenotypes and we also tested the effect of adding back wild type copies of the miRNA. For two of these; *mir-1* and *mir-80* we observed lifespan alteration in the same direction, whether the miRNA was absent or over-expressed (Supplemental Table 3 and Supplemental Figure 4). This may indicate that the miRNAs can either inhibit or promote long-life depending on the dosage. An alternative explanation may be confounding interference from background mutations that also impinge on longevity pathways.

We chose to focus further genetic characterization on the only miRNA mutant we analyzed that showed a severe lifespan phenotype (*mir-71*). *mir-71* has now been identified by multiple recent reports as having age dependent expression, being strongly required for normal longevity and being involved in germline mediated lifespan extension respectively [17, 19, 20, 22], among other phenotypes. One recent report has implicated *mir-71* as being involved in the lifespan extension derived from ablating the germline [22]. That study is difficult to compare to our current study since their genetic experiments were performed in germline deficient backgrounds and did not address the role *mir-71* has in determining wild type lifespan. Mutants of *mir-71* are profoundly short-lived, and in this respect *mir-71* is currently the single most important miRNA involved in *C. elegans* aging. Despite this, no target of the putative regulatory RNA has been established for longevity. Yet recent work has implicated it in the IIS pathway. Specifically, de Lencastre and colleagues reported that *mir-71* works with IIS and DAF-16 to determine lifespan, based on their finding that the lifespan of double mutants of *mir-71; daf-16 (RNAi)* were not enhanced for lifespan effects (short life). Contrary to this finding, we determined that double null mutants are in fact significantly shorter lived than either of the single mutants. This disparity led us to further test the model that *mir-71* works with IIS. Our results instead supported a model where *mir-71*'s impact on lifespan is at least partly independent of IIS. While we were unable to determine whether *mir-71* is completely independent of IIS pathways, our results lead us to expect that *mir-71* and IIS are likely to be mostly (if not entirely) independent. Qualitatively, the two pathways appear to be quite distinct. While the mutant phenotypes are similar for the two pathways, both lead to short life, the gain of functions are distinctly different. Over-expression of *mir-71* results in a relatively mild increase in lifespan (Figure 4A), while *daf-16* over-activation (through de-repression) can result in massive extension of lifespan [39]. Given that miRNAs are expected to act through the repression of a target mRNA's expression, we favor a model where *mir-71* is required to down regulate a target(s) that is extremely detrimental to a long life, but *mir-71* does not itself promote the expression of major stress resistance pathways, as does DAF-16. A recent report [40] has demonstrated a role for *mir-71* in recovery from starvation induced arrest, and suggested that it functions through insulin/IGF signals, but also through insulin/IGF independent pathways, as the *mir-71* recovery phenotype is enhanced by mutations in *daf-16*. This interpretation is consistent with our results, which suggest that for lifespan, the insulin/IGF independent pathway(s) of *mir-71* are the more important targets for lifespan determination. Of particular relevance is a recent report on the general involvement of miRNAs in lifespan and aging in C. elegans [41]. That study demonstrated that inactivation of miRNA synthesis in adult animas led to a major decrease in lifespan and premature aging (a phenotype very similar to *mir-71* mutants). They went on to show that the longevity of IIS mutants did not require adult miRNAs and further that the adult miRNA knockout also enhanced the short life of *daf-16* mutants [41]. That study therefore directly supports our finding that *mir-71* and IIS roles in lifespan are independent, at least in part. Many questions remain about the role of *mir-71* in aging and lifespan, future work that leads to the identification of the regulatory targets of *mir-71* will certainly help illuminate the roles of*mir-71* in lifespan determination.

miRNAs are emerging as important components of longevity pathways. Exactly which miRNAs are involved and how they impinge on known pathways will lead to a better understanding of both the biological determinants of lifespan and the mechanisms of miRNA modulation of aging.

## METHODS

### Nematode Strains

Nematodes were maintained as previously described [42]. The following previously described alleles, strains and transgenics were used in the experiments presented here. N2; *glp-1(e2141)*; daf-16(mu86); daf-2(e1368); mir-71(n4115); maIs352[Pmir-71::GFP]; *mir-85(n4117); mir-42-44(nDf49); mir-84(n4037); mir-273(n4438); mir-51(n4473); mir-54-56(nDf58); mir-240(n4541); mir-60(n4947); mir-70(n4109); mir-81-82(nDf54); mir-53(n4113);mir-235(n4504);nDf63; mir-63(n4568);mir-80(nDf53);mir-64c(nDf63);mir-1(n4101); mir-58(n4640);mir-228(n4382); maIs251[Pmir-1::GFP];* maIs276[Pmir-60::GFP]; maIs188[Pmir-228::GFP].

### Lifespan Assays

Lifespan assays were performed as previously described [43]. In brief, synchronized worm populations were transferred to NGM plates supplemented with 10μg/mL floxuridine (FUDR) at day one of adulthood. Animals were scored and transferred to fresh plates approximately every other day until death. Lifespans presented in Figure 1 and Figure 3 did not contain FUDR nor did any lifespan that involved the mutant *glp-1*. Lifespan assays containing *glp-1* were shifted to 25°C immediately after the egg lay and maintained at that temperature, all other lifespan assays were performed at 20°C. Comparisons of survival were performed with GraphPad Prism 4™ software and P values were generated by the log-rank Whitney test of significance.

### Preparation of samples for BioMark

To quantify changes in miRNA abundance during aging, we cultured worms under standard laboratory conditions and aged them by daily transferring the adults away from their progeny. At selected ages, we harvested individual animals and isolated total RNA from each sample using the miRNeasy (Qiagen) collection kit with automated Qiacube processing (Qiagen). cDNA synthesis was accomplished with the high capacity RT kit (Applied Biosciences). miRNA reverse transcription was performed with Applied Biosciences custom made miRNA reverse transcription primers. Reverse transcription products were subjected to a multiplex pre-amplification prior to the qPCR.

### miRNA Detection by Quantitative Polymerase Chain Reaction

qPCR assays for 69 miRNAs were obtained from Applied Biosystems (ABI), at the time this represented their entire coverage of *C. elegans* miRNAs. Currently over 170 miRNAs are listed on miRBase (www.mirbase.org) as existing in the C. elegans genome. However, the validity of this estimate as being representative of the actual number of miRNAs is unclear. A recent report using high-throughput sequencing techniques revealed that of the approximately 170 annotated miRNAs (at the time) only 106 carried nucleotide signatures consistent with processing by the miRNA processing enzymes [44]. qPCR on the BioMark (Fluidigm) was performed according to the protocol of [31] with slight modifications for use with single *C. elegans* samples.

Additional methods are included with the Supplemental information.

## SUPPLEMENTARY DATA


