# Supplementary material for: Age-related micro-RNA abundance in individual C. elegans
Source: Aging (Albany NY). 2013 Jun 12;5(6):394–411. doi: 10.18632/aging.100564 (PMC3824409; doi:10.18632/aging.100564)
Supplement: Supplementary file 1 [file aging-05-394-s001.pdf]

elegans from starvation-induced L1 diapause. Proceedings of the National Academy of Sciences of the United States of America. 2011; 108:17997-18002.

41. Lehrbach NJ, Castro C, Murfitt KJ, Abreu-Goodger C, Griffin JL and Miska EA. Post-developmental microRNA expression is required for normal physiology, and regulates aging in parallel to insulin/IGF-1 signaling in C. elegans. RNA. 2012; 18:2220-2235.

42. Brenner S. The genetics of Caenorhabditis elegans. Genetics. 1974; 77:71-94.

43. Lucanic M, Held JM, Vantipalli MC, Klang IM, Graham JB, Gibson BW, Lithgow GJ and Gill MS. N-acylethanolamine signalling mediates the effect of diet on lifespan in Caenorhabditis elegans. Nature. 2011; 473:226-229.

44. Warf MB, Johnson WE and Bass BL. Improved annotation of C. elegans microRNAs by deep sequencing reveals structures associated with processing by Drosha and Dicer. RNA. 2011; 17:563-577.

SUPPLEMENTARY MATERIALS

Supplementary Methods

Normalization and Statistical Analysis of miRNA Profiling Results. The following steps were taken to estimate the ratio of expression of miRNAs among samples of different ages in the WT and glp-1 mutant experiments. The only important difference among these two with regards to the analysis is the number of different ages at which the miRNA were examined: 2 for WT (day 12 vs. 1) and 4 for glp-1 mutants (days 1, 6, 12, and 18). For both, the results are based on linear models, but in the glp-1 experiment, this means that the parameter of interest was the average rate of change in expression over these different days. Both the WT and the glp-1 experiments consisted of two different plates (with distinct sets of miRNA's), and the analysis of these was done equivalently. First, we derived the mean ct count among the technical reps for each sample, miRNA, which generally were 2-4. Second, with a structure now of miRNA's versus mean ct's for each sample (forming a matrix of number of miRNA's vs. samples), we used loess normalization (Bolstadt et al. 2003). As there can be no missing values for this normalization as implemented, before normalization we imputed missing values using nearest neighbor techniques (Troyanskaya et al. 2001). After normalization, missing values were set back to missing (the imputed values do not contribute to the analysis, only to aiding in the normalization, and results not sensitive to tuning parameters of imputation). Normalization was done because of very obvious large sample to sample differences in the overall distribution of within sample expression of the miRNAs, thus attempting to remove nuisance variance caused by the inability to control certain experimental factors. Each of the plates (sets of miRNA's) was normalized separately, resulting in two sets of sub-experiments, but each with its own set of miRNA's. We conducted simple linear regression of the mean ct per sample i, for miRNA j (say Yij) to get the estimate of E(Yij|Agei) = aj+bj*Agei, so bj represents the mean change in ct for a change in Age: we equated the results of the two experiments, by converting this to a change of 11 days. We estimate differential expression of the miRNA j (treating relative reduction of one ct as a doubling in the abundance of the miRNA's), as: , where c is based on the scale of the age variable entered in the model (c=1 for WT, and c=11 for glp-1). We derived the inference using robust (sandwich) variance estimates of the bj, so our inference is robust against violations of the standard normal linear model assumptions. We report both the raw p-value and the adjusted p-value, adjusted for multiple comparisons (family-wise error rate) via the standard and conservative Bonferroni correction).

Generation of transgenic nematode strains. All transgenic animals for this study were generated through gonad microinjection. To create the Phsp-16.2::mir-71 construct with mir-71 under the hsp-16.2 promoter, a 325 bp genomic fragment containing the hsp-16.2 promoter was amplified from N2 genomic DNA by a polymerase chain reaction (PCR) (primer F1: GGAGAACGGAGCATGAGCATTTT; primer R1: TGTTGTTCGGTATTTATTTTCAACGGTATTTATA CTA). This fragment was fused to another genomic DNA fragment which contained the DNA sequence for the entire mir-71 pre-micro RNA (primer F1: TAGTATAAATACCGTTGAAAATAAATACCGAA CAACATCACACACAGAGGTTGTCTGCTC; primer R1: ATTGAGCCGATGACTGGAAGGAACA) as well as a 5' end with complimentary sequence to the hsp-16.2 promoter sequence. The PCR fusion (Horton et al. 1989; Hobert 2002) was performed with the primers F1: AAGCGTGTTGGCTTCCTCTGAGC and R1: AGTTCAATTCACAACGTGATCCTGTG.

To generate the Pmyo-2::mir-71 construct the same strategy was used. The primers F1: GCGTGCGGAGG TTTAGAGAAGGA and R1: TTCTGTGTCTGACGAT CGAGG were used to create a PCR fragment containing the myo-2 promoter. This was fused to a PCR fragment containing mir-71 (created in the same manner as described above except with a different 5' end which was complimentary to the myo-2 promoter sequence primer F1:CATTTTAACCCTCGATCGTCA GACACAGAATCACACACAGAGGTTGTCTGCTC). The PCR fusion was performed with primers F1: GGTGGTGGACAGTAACTGTCTGT and R1: AGTT CAATTCACAACGTGATCCTGTG.

We utilized PCR to generate rescue fragments for several candidate miRNAs. Transgenic worm strains expressing PCR amplified genomic DNA fragments were generated through gonad microinjection. Genomic miRNA fragment constructs were injected into the gonad at a concentration of 1, 2 or 10 ng/µL. Injection mix was brought to a final concentration of 100 ng/µL by supplementation of co-injection marker(s). When possible the upstream primer was designed such that they overlapped or were 5' to previously reported promoter regions, while the 3' primer was always located downstream of the end of the pre-miRNA. Pre-miRNA sequences were obtained from www.mirbase.org. mir1.f1 ctagacacgctgacagtttgcca; mir1.r1 ttgaacggtgagagaggatcgga. mir64c.f1 ggcttctgggagtctgagccaat; mir64c.r1 ctgcaatcaatacgagcagttgcagg. mir67.f1 gcttcatgctcgtatagggttctgag; mir67.r1 cgtgaatagcgtgttctcagcattc. mir80.f1 gtgacgtcaaagatggtcgccgc; mir80.r1 ctccaggagaatgtgatccgaac. mir81.f1 gccacaagtacagtttggcaacatcg; mir81.r1 gggatccgaatatgcgacagtgc. mir82.f1 ggacatattctcaacaacgtagcccg; mir82.r1 gcgcaactcctgtatcggttagc. mir228.f1 gcgggaagagacgagattactgt; mir228.r1 aaatggccatgggaaaggcgctc. mir235.f1 gacatgcatgtactgcggctgct; mir235.r1 ggatcggccatcagaacagtgca.

Microscopy. Worms were mounted on cover slips atop a 2% agarose pad and anaesthetized with 3µM levamisole prior to imaging. Images were captured with an Olympus BX51 compound microscope equipped with a Hamamatsu Orca-ER camera. Image analysis was performed with Image J software (NIH).

Northern Blot. Total RNA was extracted from an approximately 100µL pellet of mixed stage C. elegans collected from mass culture nematode growth media agar plates (Lucanic et al 2011). RNA was prepared as described above for QPCR experiments. Probes for the northern blot for the daf-2 coding sequence were generated with primers E14.f1: CGAGATCTCGCCGC ACGTAATTG and E14.r1: CAACGACTCGGGTGAC ATCCATC and for the mir-71 binding site in the 3' UTR the primers were UTR.f1: CCCACATTATCATA TCTCTACACGAATATCGG and UTR.r1: TGAAAG ACACTATCTATGGAATTCACGGG.

Supplementary References

1. Bolstad BM, Irizarry RA, Astrand M, Speed TP. A comparison of normalization methods for high density oligonucleotide array data based on variance and bias. Bioinformatics. 2003; 19:185-193.

2. Hobert O. PCR fusion-based approach to create reporter gene constructs for expression analysis in transgenic C. elegans. Biotechniques. 2002; 32:728-730.

3. Horton RM, Hunt HD, Ho SN, Pullen JK , Pease LR. Engineering hybrid genes without the use of restriction enzymes: gene splicing by overlap extension. Gene. 1989; 77: 61-68.
Rehmsmeier, M., Steffen, P., Hochsmann, M., and Giegerich, R. Fast and effective prediction of microRNA/target duplexes. RNA. 2004; 10:1507-1517.

4. Troyanskaya O, Cantor M, Sherlock G, Brown P, Hastie T, Tibshirani R, Botstein D , Altman RB. Missing value estimation methods for DNA microarrays. Bioinformatics. 2001; 17:520-525.

Supplementary Tables

Supplemental Tables of this manuscript are found in full version at www.impactaging.com/papers/v5/n6/full/100564.html.

Supplementary Figures

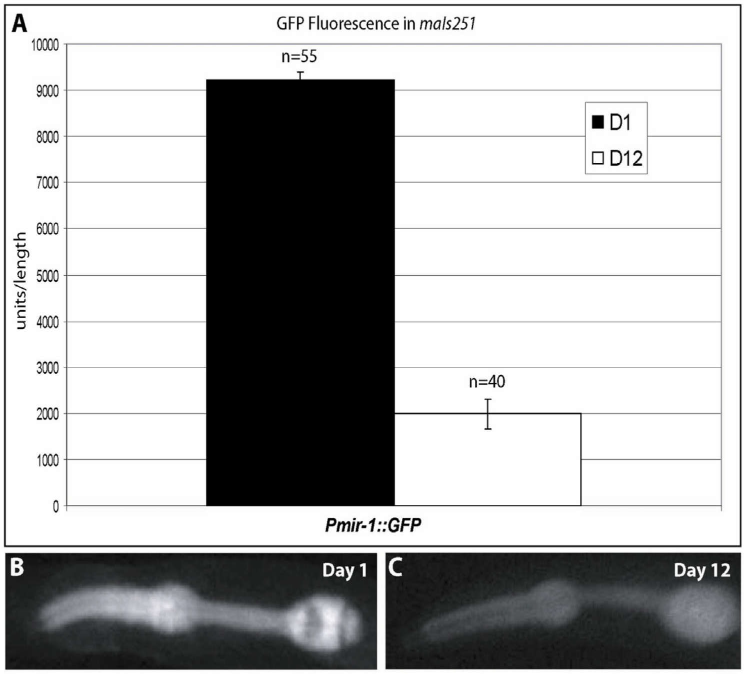

Supplemental Figure 1. GFP fluorescence expressed from a *mir-1* promoter-GFP fusion decreases with age. (**A**) Graphical representation of GFP expression in the *maIs251[Pmir-1::GFP]* strain, VT1665. Images were acquired at the same magnification and exposure. To circumvent intestinal auto-fluorescence that accumulates with age image fluorescence was only measured in anterior structures. (**B-C**) Example images and region used for analysis demonstrating the decrease in fluorescence between young (**B**) and old (**C**) animals.

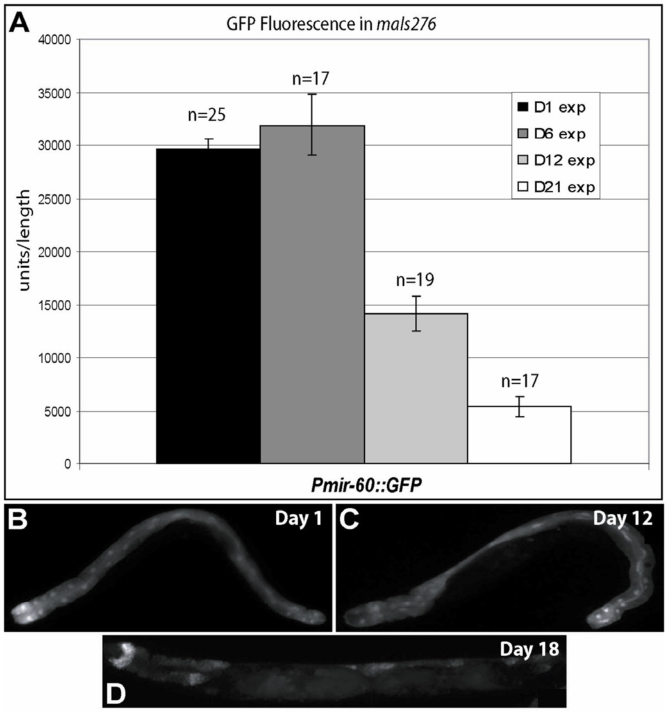

Supplemental Figure 2. GFP fluorescence expressed from a *mir-60* promoter-GFP fusion decreases with age. (**A**) Graphical representation of GFP fluorescence in the *maIs276[Pmir-60::GFP]* strain, VT1733. Images were acquired at the same magnification and exposure. This strain strongly expresses GFP exclusively in the intestine and so the exposure time used to capture the fluorescence images was brief. This allowed for some mitigation of the confounding increase in intestinal auto-fluorescence with age, since N2 controls had low or no fluorescence signals when imaged at the same exposure (data not shown). However, the decrease in signal with age shown for the reporter strain may be under represented due to increasing auto-fluorescence with age. (**B-C**) Example images and region used for analysis demonstrating the decrease in fluorescence between young (**B**) and old (**C**) animals.

.

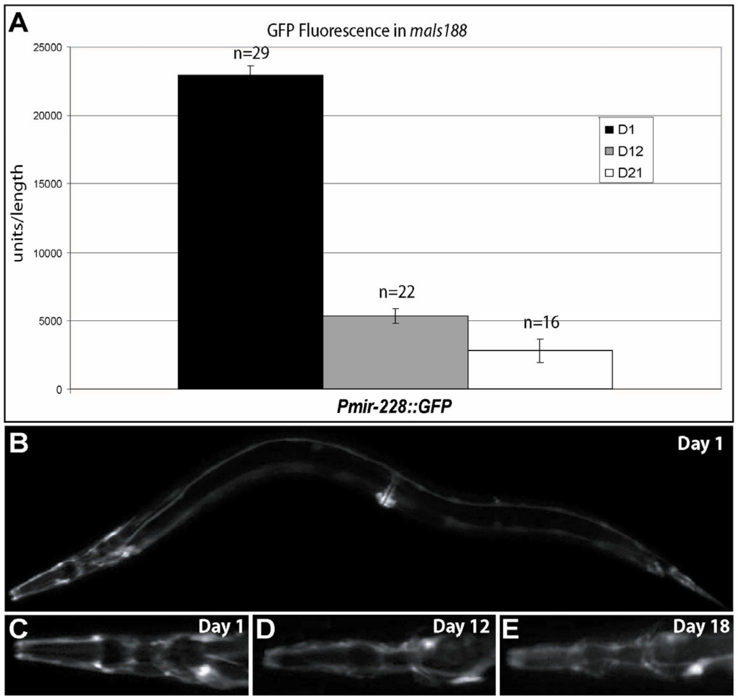

Supplemental Figure 3. GFP fluorescence expressed from a *mir-228* promoter-GFP fusion decreases with age. (**A**) Graphical representation of GFP expression in the *maIs188[Pmir-228::GFP]* strain, VT1485. Images were acquired at the same magnification and exposure. To circumvent intestinal auto-fluorescence that accumulates with age image fluorescence was only measured in anterior structures. (**B-E**) Example images of the reporter strain used for analysis, showing both an entire young adult (**B**) and the anterior region used for analysis (**C-E**), demonstrating the decrease in fluorescence between young (**C**) and old (**D-E**) animals.

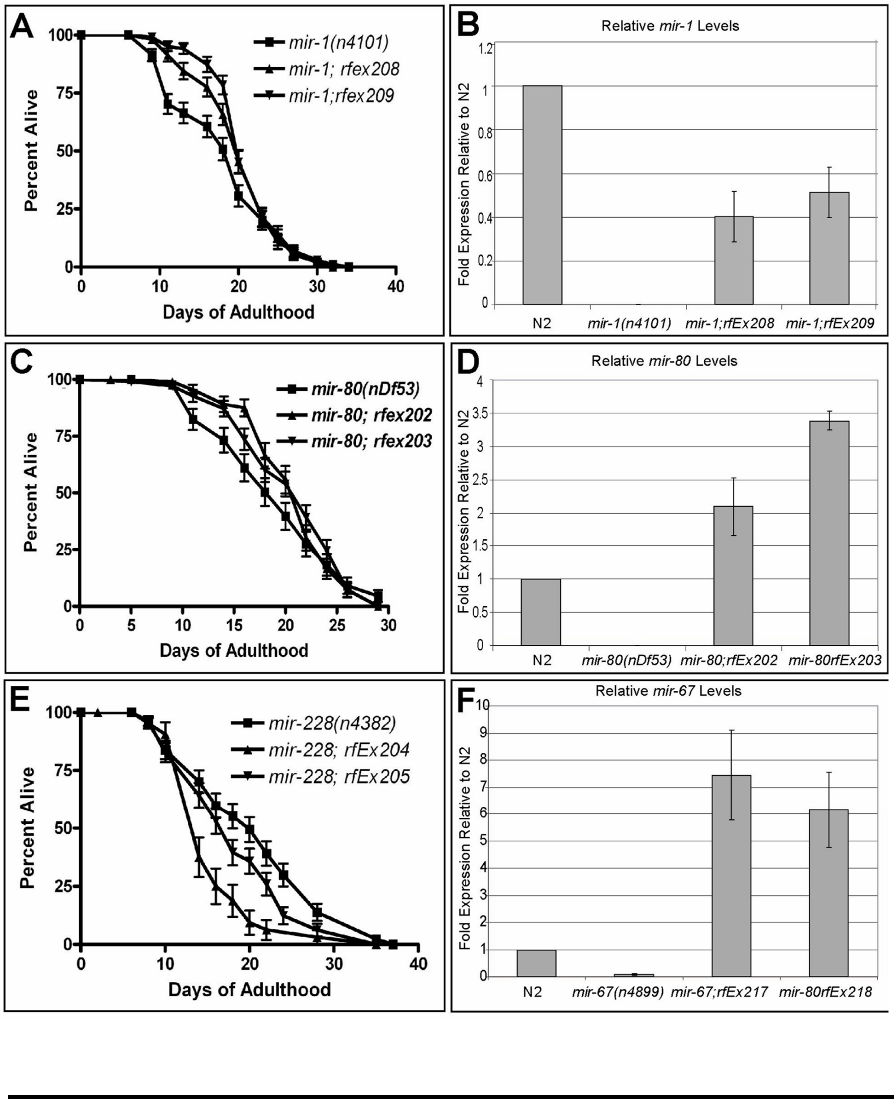

Supplemental Figure 4. Expression of miRNAs, modulates miRNA mutant's lifespan phenotypes. Transgenic *mir-1* mutants containing the *mir-1* gene in an extra-chromosomal array (*rfEx208-209[Pmir-1::mir-1]*) showed an extended lifespan compared to their non-transgenic siblings (**A**). These transgenic lines expressed *mir-1* miRNAs at a level approximately half that of wild type (**B**). Transgenic *mir-80* mutant animals containing the *mir-80* gene in an extra-chromosomal array (*rfEx202-203[Pmir-80::mir-80]*) had an extended median lifespan relative to their non-transgenic siblings. The transgenic mutant lines, *rfEx202* and *rfEx203* were found to express *mir-80*, 2 and 3.5 fold higher than wild type animals (**D**). Expression of *mir-228* was found to reduce the lifespan of the *mir-228* mutants (**E**). Expression of *mir-67* in the *mir-67* mutant transgenic lines rfEx217 and rfEx218 was determined to be approximately 6 fold higher than in wild type animals (**F**). Lifespan assay graphs are from representative experiments and a complete summary of lifespan experiments is presented in Supplementary Table 3. For miRNA rescue experiments, miRNA levels were determined by quantitative reverse transcriptase PCR from triplicate biological samples and were normalized to *rps-0* mRNA levels and are shown relative to wild type levels.

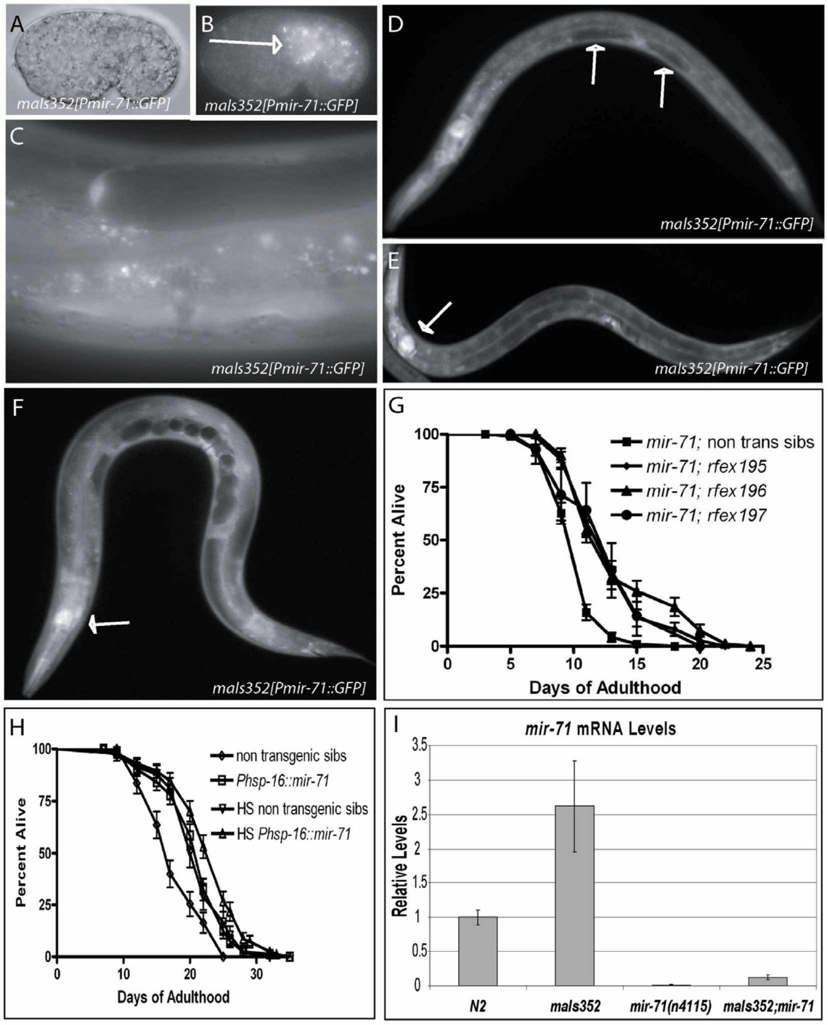

Supplemental Figure 5. The trans-gene *maIs352* expresses both GFP and the miRNA *mir-71.* (A-F) Expression of GFP driven by the *mir-71* promoter is observed specifically in the intestine primordial cells of the comma stage embryo (**A-B**) and is present in the adult as well (**C**). Post hatching, expression was observed in most somatic tissues (**D**). Arrows in (**D**) indicate the elongating gonad. Expression was also high in the pharynx in developing (**E**) and adult animals (**F**). (**E-F**) Arrows denote the pharynx. In all images anterior is to the left and dorsal is up. (**G**) Pharyngeal expression of *mir-71* partially rescues the *mir-71* mutant. Survivorship plot from *mir-71* and *mir-71; rfEx[Pmyo-2::mir-71]* mutant animals. *rfEx195-197* are 3 independent lines expressing *[Pmyo-2::mir::71]*. (**H**) Over-expression of *mir-71* significantly extends the lifespan of otherwise wild type animals. (**I**) A graphical representation of the averaged, relative levels of *mir-71* in several different strains (staged at L4) as determined by quantitative PCR. *mir-71* levels were normalized to *gpd-1* (GAPDH). Error bars reflect the standard error of the mean and the data was derived from three biological replicates with three technical replicates for each qPCR.

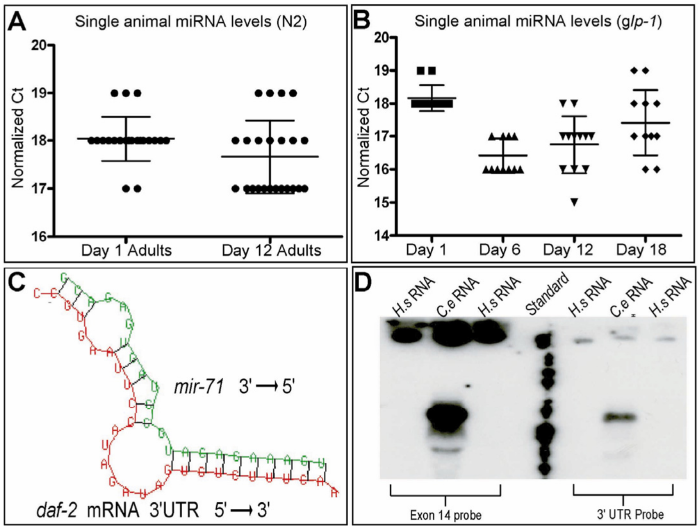

Supplemental Figure 6. (**A-B**) Normalized Ct values describing *mir-71* abundance in individual worms (each point represents data from a single animal). Normalized Ct values for *mir-71* wild type (N2) animals at two ages shown in (**A**) and *glp-1* mutant animals at four ages shown in (**D**). (**C**) Schematic showing a predicted interaction between *mir-71* and the *daf-2* 3'UTR. Prediction was generated with RNAhybrid (Rehmsmeier et al. 2004) (**D**) Northern blot showing wild type total RNA fractions treated with radioactive probes for the coding region (exon 14) or the 3' UTR (*mir-71* binding site). Probe level was not equivalent as can be seen from the signal in the well.
